# The valorization of municipal grass waste for the extraction of cellulose nanocrystals

**DOI:** 10.1039/d0ra07972c

**Published:** 2020-11-23

**Authors:** Wan Hazman Danial, Raihan Mohd Taib, Mohd Armi Abu Samah, Rosliza Mohd Salim, Zaiton Abdul Majid

**Affiliations:** Department of Chemistry, Kulliyyah of Science, International Islamic University Malaysia 25200 Kuantan Malaysia whazman@iium.edu.my; Department of Chemistry, Faculty of Science, Universiti Teknologi Malaysia 81310 UTM Johor Bahru Johor Malaysia

## Abstract

The study reports on the valorization of municipal grass waste (MGW) for the extraction of cellulose nanocrystals (CNCs), as an eco-friendly and sustainable low-cost precursor for cellulose nanomaterial production. The raw MGW was subjected to boiling in water pretreatment, and alkali and bleaching treatments for the extraction of cellulose fibers, followed by isolation of the CNCs through a conventional acid hydrolysis technique. Fourier transform infrared spectroscopy was used to analyze the cellulose fibers extracted while scanning electron microscopy and transmission electron microscopy images confirmed the presence of cellulose fibers and CNCs, respectively. The chemical composition of MGW was ascertained through the TAPPI-222 om-02 standard for lignin content and determination of α-cellulose. The diameters of CNCs are in the range of 5–15 nm with the length ranging from 100 nm to 500 nm, while a crystallinity index of 58.2% was determined from X-ray diffraction analysis. The production of CNCs from MGW is an avenue to convert green waste into a value-added product, in addition to reducing the volume of cumulative waste in the environment.

## Abstract 2

The production of CNCs from MGW is an avenue to convert green waste into a value-added product.

## Graphical abstract

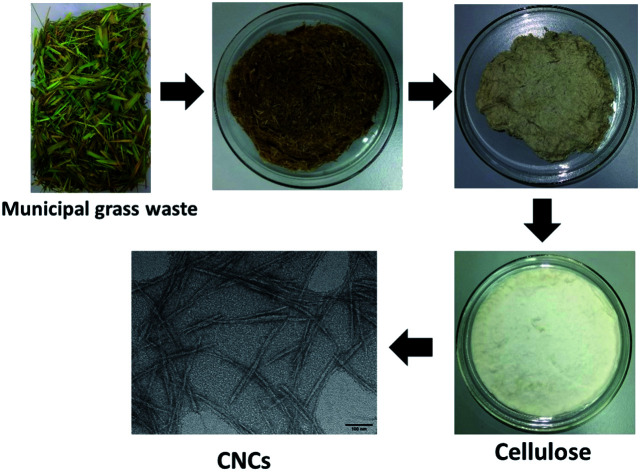

## Introduction

Over the last few decades, cellulose nanomaterials have shown tremendous application prospects due to their remarkable chemical and physical properties including high tensile strength, good elastic modulus of approximately 150 GPa, large specific surface area of up to several hundred m^2^ g^−1^, and low density of around 1.61 g cm^−3^.^1,2^ In addition, the presence of reactive surfaces, combined with renewability, sustainability and biodegradability, further expands its applicability.^2–4^ The method of preparation, dimensions and functional capabilities of the cellulose nanomaterials are very much dependent on the source material and processing conditions.^5^ Cellulose nanomaterials that are extracted from lignocellulosic biomass can be classified into two main subcategories: (i) cellulose nanofibers (CNFs), which are elongated fibrils comprising both amorphous and crystalline regions,^6^ and (ii) cellulose nanocrystals (CNCs), which are highly crystalline nanoparticles of cellulose prepared *via* strong acid hydrolysis.^7,8^

CNCs or sometimes referred as cellulose nanowhiskers commonly have length of several hundred nanometers and diameters ranging from 1 to 50 nm,^9^ which makes them compatible in developing nanocomposites and high-performance composites^10–12^ due to their high crystallinity and high surface area. Besides, the intriguing and engineered properties of CNCs makes it an ideal material to be considered in niche fields of applications including but not limited to biomedical,^13^ food packaging,^14^ reinforcing fillers,^15^ membranes,^16^ optical^17^ and environmental.^18,19^

CNCs are generally produced by hydrolyzing the amorphous or semicrystalline fraction of cellulose, which are very susceptible to strong acid treatment. Therefore, acid hydrolysis is the conventional and commonly applied procedure for the production of CNCs,^20,21^ only slightly differentiated with a small interval of temperature and duration of acid hydrolysis. Despite can be derived from various sources; the selection of the cellulose precursor for the extraction of CNCs has been actively investigated. As commonly reported in the literature, the extraction of CNCs were usually carried out by utilizing cellulosic-based materials such as waste papers,^22–26^ wood pulps,^27,28^ cellulose powder,^29^ microcrystalline cellulose,^30^ cotton,^31^ hemp,^32^ and sisal.^33^ However, all these sources have their significant manufacturing cycle in production of cellulose or recycling industries.

This work focused on the valorization of municipal grass waste (MGW) as a promising source material for the extraction of CNCs. The MGW is also known as grass clippings, and considered as a green waste, which is a major constituent of solid waste.^34^ The green waste management is rather complex and expensive due to its constant generation and occupies large volume.^35,36^ Albeit an alternative on composting the green waste has also been considered, the presence of recalcitrance component such as lignin and cellulose increase the processing time, thusly hamper the effort.^37^ The limitations of the composting efforts include long processing time, low product quality and require a proper and optimum strategy.^37^ The piled-up MGW is not only unsightly, but also contributes to the problem of municipal solid waste disposal and flooding landfills. The MGW is an accumulated waste that has no significant commercial uses or industrial importance. Besides, it is usually left untreated and accumulated as stockpile without any specific use afterwards or in worst-case scenario, it was collected and burned, which inevitably contributes to open fire burning, air pollution and global warming. Thus, an avenue to convert the green waste into value-added product would be significant so as to ensure sustainable consumption; adapting cradle to cradle concept. Therefore, it is worth mentioning that valorization of such green waste material is a favorable approach for the extraction of CNCs without impeding with the commercially or widely used cellulosic-based material sources. Various other sources of agro-waste materials for the production of CNFs or CNCs have also been reported such as sugarcane bagasse,^38^ rice straw,^39^ oil palm empty fruit bunch,^40^ apple pomace,^41^ cucumber peels,^42^ tomato peels,^43^ pineapple peels,^44^ and royal palm tree.^45^

In addition, the accessibility for the isolation of cellulose fibers from grass waste is an alternative initiative to other commonly used precursors. Although the wood is the preferred source of cellulose due to its availability, the wood fiber costs ranged between 38–45% of the total production cost and the feedstock cost is among the major cost drivers.^46^ Despite there is no available information for the industrial production cost of cellulose from grass waste, it is expected that the plant fiber cost derived from the grass clippings would be lower based on the fact that it is a waste utilization. Besides, a huge increase in papermaking and cellulose production, both which are traditionally sourced from wood pulp, has resulted in the severe exploitation of trees, thereby leading to environmental problems such as deforestation. To mitigate the deforestation impact, the valorization of the grass waste can be a significant alternative approach.

To the best of authors' knowledge, the extraction of CNCs from the MGW or grass clippings has not been reported yet. In this study, cellulose fibers were isolated from raw MGW using several treatment processes and followed by controlled-condition of acid hydrolysis for the extraction of CNCs. Structural analysis and characterization were carried out using Fourier transform infrared (FTIR) spectroscopy, scanning electron microscopy (SEM), transmission electron microscopy (TEM), and X-ray diffraction (XRD) analysis.

## Experimental

### Materials

The raw material of the MGW used in this study was sourced from a stockpile of grass waste produced from post lawn-mowing activity located in the city of Kuantan, Pahang, Malaysia. Sodium hydroxide, NaOH (R&M Chemicals), hydrogen peroxide, H_2_O_2_ (Merck), sodium chlorite, NaClO_2_ (R&M Chemicals), and sulfuric acid, H_2_SO_4_ (Merck) were used as received without further purification.

### Extraction of cellulose fibers

#### Pre-treatment

25 g of the collected MGW were weighed and boiled in 500 mL distilled water for 60 minutes. The filtered MGW was then ground using mechanical grinder and re-boiled in 500 mL distilled water for another 60 minutes. The sample was then filtered and rinsed several times with distilled water. The pretreated sample was completely dried in an oven at 80 °C for 90 minutes. The dried pretreated sample is labelled as PTMGW.

#### Alkali treatment

Extraction of cellulose was further conducted through alkaline treatment by eliminating lignin and hemicellulose from pretreated MGW. The PTMGW was treated and boiled with 4 wt% NaOH solution under reflux system for 90 minutes. The alkali treated sample was then filtered and washed with distilled water to remove any excess NaOH until a neutral pH was achieved. The sample was then completely dried in the oven at 80 °C for 90 minutes. The dried alkali treated sample is labelled as ATMGW.

#### Bleach treatment

For the bleaching treatment, the procedure was adapted from Candido and Gonçalves^47^ for the isolation and purification of cellulose fibers, whereby the ATMGW was subjected for bleaching using 5% H_2_O_2_ (v/v), 1.3% NaOH (m/v), and 0.7% NaClO_2_ (w/v) under reflux condition at 80 °C for 90 minutes. The bleached MGW was filtered and washed until neutral pH was achieved. The bleaching treatment was repeated for another 60 minutes to ensure the complete removal of lignin or any undesirable components. The bleached sample was then filtered and washed again until a constant pH was achieved. The bleach treated MGW was then completely dried in the oven at 60 °C for 60 minutes. The dried samples for the first bleaching treatment and the second bleaching treatment were labelled as BT1MGW and BT2MGW, respectively.

### Isolation of cellulose nanocrystals (CNCs)

#### Acid hydrolysis

5 g of the dried BT2MGW were weighed and suspended with 10 mL of distilled water. The suspended BT2MGW was then reacted with 64% H_2_SO_4_ with a ratio of 3 : 8 (w/v) under constant vigorous stirring for 60 minutes at 45 °C. The reaction was halted with the addition of distilled water 10 times to the volume of acid used. The hydrolyzed sample was subjected to centrifugation (5300 rpm, 10 minutes) to remove the excess sulphuric acid. The sample was then dialyzed against distilled water until a constant pH was achieved.

### Characterization of cellulose and CNCs

#### Fourier transform infrared (FTIR) spectroscopy

The FTIR analysis (PerkinElmer Frontier) was carried out in the range of 4000–400 cm^−1^ to identify the lignocellulosic components present in the samples. The dried MGW, ATMGW, BT1MGW and BT2MGW samples were ground and blended with KBr powder before pressing the mixture into ultra-thin pellets.

#### Scanning electron microscopy (SEM)

The morphology of dried raw MGW, ATMGW, BT1MGW and BT2MGW were examined by Zeiss Evo scanning electron microscope. The dried samples were deposited on a metallic stub using carbon tape and gold coated to avoid sample charging, using a sputter coater. The SEM analysis was operated under high vacuum mode, with an acceleration voltage between 10–20 kV.

#### Transmission electron microscopy (TEM)

TEM images of the CNCs were recorded using TEM (Hitachi HT7700, Japan) with an acceleration voltage of 100 kV. The sample was subjected for 4 hours of magnetic stirring followed by ultrasonication for 30 minutes. A drop of the CNCs suspension was deposited on copper grid and was then dyed using UranyLess staining solution for 5 min.

#### X-ray diffraction (XRD)

XRD analysis was carried out using a Phillip PW 3040/60 MPD X'Pert High Pro PANalytical diffractometer with Cu Kα radiation, between 2*θ* of 5–40°, with a 2*θ* step size of 0.008° and a time of 20 s per step. The crystallite size was estimated from the FWHM (full width at half maximum) value of the 100% cellulose peak around 22.3°, using the Scherrer equation:1
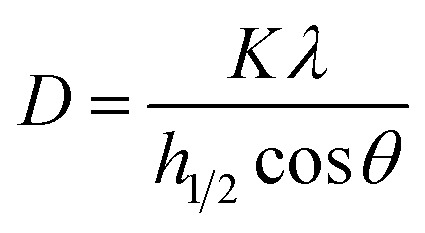
where *D* = average size of the crystallites, *K* = Scherrer constant (0.94 for spherical crystals), *λ* = wavelength of radiation (0.154 nm), *h*_1/2_ = FWHM, and *θ* = Bragg angle (the peak position = 2*θ*). The crystallinity index (CI) was calculated using eqn (2).^48,49^2
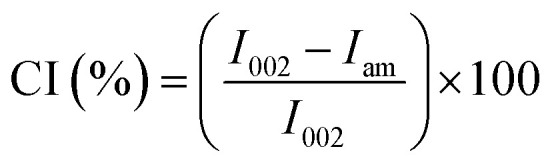
where *I*_002_ is the maximum diffraction intensity of the peak of 2*θ* around 22°–23° while *I*_am_ is the diffraction intensity of the 2*θ* between 15° and 16° which represents the amorphous background, which is the minimum diffraction intensity for cellulose.^38,50^

### Composition of municipal grass waste (MGW)

#### Lignin content

The procedure for lignin determination was carried out according to the TAPPI-222 om-02 standard; whereby 72 mL of 72% (v/v) H_2_SO_4_ was mixed with 2 g of the raw MGW in a round bottom flask with constant magnetic stirring for 2 hours at room temperature. Subsequently, 560 mL of distilled water was added and the mixture was heated to the boiling point, followed by refluxed for 4 hours. After cooling, the mixture was subjected to vacuum filtration and dried in the oven at ∼100 °C for 2 h before it was weighed for the lignin content. The procedure was performed in triplicate.

#### α-Cellulose determination

50 mg of the obtained cellulose was weighed and placed in a beaker. Then, 4 mL of 17.5% (w/w) NaOH was added and left aside to react for 300 min at room temperature. Subsequently, 4 mL of distilled water was added and the mixture was stirred with a spatula for 1 min and left to rest another 30 minutes. The suspension was filtered and washed thrice with 30 mL of distilled water each, and moistened with an acetic acid solution for 5 min. The neutralized fibers were washed again thrice with 30 mL distilled water each and the amount of α-cellulose was determined when the sample was completely dried in the oven at 100 °C for 4 hours. The procedure was performed in triplicate.

## Results and discussion

### Isolation of cellulose fibers

**Fig. 1:**
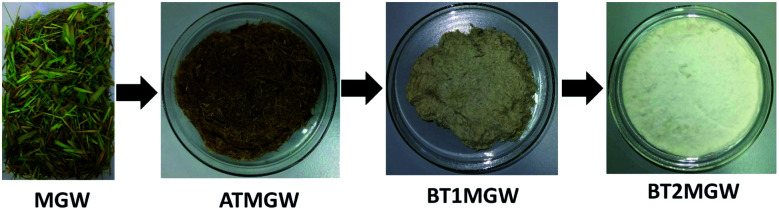
Digital images of raw MGW, ATMGW, BT1MGW and BT2MGW obtained from the different treatment stages.

The alkaline treatment was conducted to isolate, as well as enhance, the cellulose quantity in the sample. This process serves to reduce the non-cellulosic portions of the fibre, which includes lignin, hemicelluloses, pectin, waxes, oils, and other contaminants.^51^ A combination of hydrogen peroxide and sodium chlorite was used during the bleaching treatment, for the removal and fractionation of a considerable quantity of residual hemicelluloses and lignin, persisting in the grass waste sample. Hydrogen peroxide reactivity gives rise to the development of an exceedingly reactive strain of superoxide radicals (O_2_˙^−^). These superoxide radicals promote the oxidation of the aromatic rings of lignin, and segments of hemicellulose for carboxylic acids.^45^ The bleaching treatment process facilitates the removal of impurities, from the fibre of the raw material, through the elimination of the chromophore groups. This process led to the realization of a whiter and less contaminated material as can be seen in Fig. 1.

In terms of chemical composition, the raw municipal grass waste (MGW) registered a lignin measure of 9.2 ± 0.4%, and 52.7 ± 0.07% of α-cellulose. Following purification, the bleached fibre (BT2MGW) registered a 58.4 ± 0.07% of α-cellulose, with only a 0.01% of lignin content. This revealed the combination of H_2_O_2_, NaOH, and NaClO_2_ for the bleaching treatment allow complete removal of lignin from the MGW. Yan *et al.*^52^ reported that efficient removal of lignin from grass waste can also be achieved using a dilute NaOH supplemented with H_2_O_2_. The FTIR analysis verified a considerable dip in the quantity of lignin, after bleaching. The significance of the bleaching treatment, for raw material purification, is associated to the need for lignin reduction. As lignin is insoluble at the sulphuric acid concentration in use, it can intervene and curtail the extraction of cellulose nanocrystals.^45^

### FTIR analysis

**Fig. 2:**
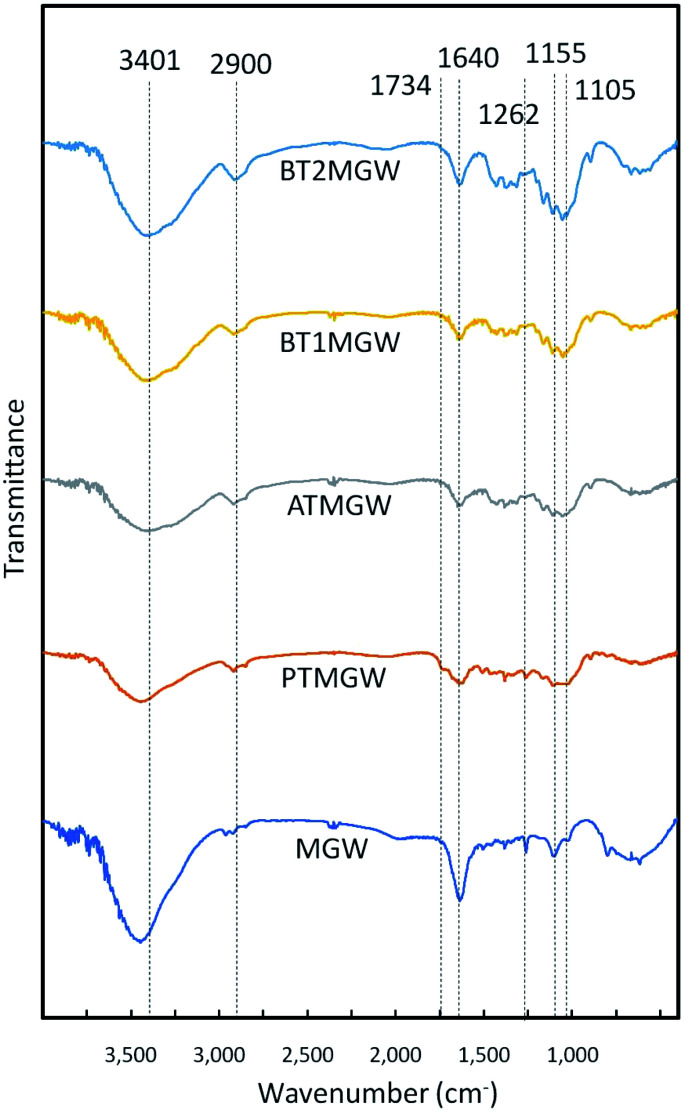
FTIR spectra of MGW, PTMGW, ATMGW, BT1MGW and BT2MGW.

The FTIR analysis (Fig. 2) revealed alterations in the properties of the materials, following the delignification of the MGW. Peaks around 3440 to 3400 cm^−1^ zone, was observed in all spectra, representing the C–H and O–H groups, associated to the cellulose component.^45^ This is an indication that the cellulose content was preserved throughout the chemical treatments performed. Peak at 2900 cm^−1^ derives from the C–H stretching vibration corresponds to general organic content.^53^ As anticipated, this circumstance is similar for all the samples.

The reduction in strength of the C=O band at 1734 cm^−1^, denoting the carboxyl groups, brought about by the acetyl group and the ester of the hemicelluloses, or the links between esters of the carboxyl groups of lignin and/or hemicellulose, implies the disbanding or elimination of hemicellulose in the samples.^54^ The peak at 1640 cm^−1^, which is apparent in all the samples, concerns the water absorption factor.^51^ Absorption between 1260 and 1274 cm^−1^, particularly at 1262 cm^−1^, relates to the elongation of the C–O bond of the aryl group.^55^ It is inclined to vanish or decline in strength following alkaline and bleaching processes. This suggests the reduction of lignin and hemicellulose during the purification stages.^13^ While a reduction of this band is apparent in the spectrum, it is nevertheless more accentuated in the raw MGW and BT2MGW spectrum, suggesting the effectiveness of alkali and bleaching treatment. The peaks at 1155 cm^−1^ and 1105 cm^−1^ respectively correspond to C–C ring breathing band and C–O–C glycoside ether band due to the presence of cellulose.^53^ Their rise in intensity may be attributed to the boost in cellulose content of the sample, after bleaching.

### SEM analysis

**Fig. 3:**
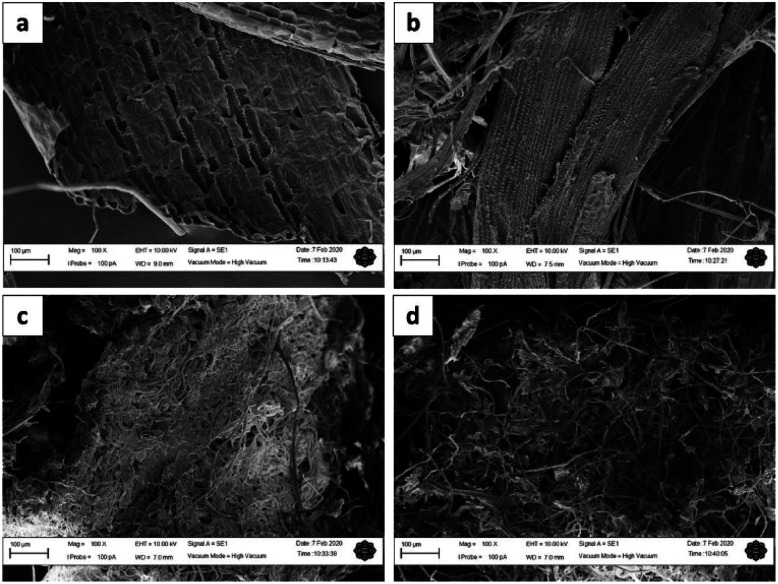
SEM micrographs of (a) MGW, (b) ATMGW, (c) BT1MGW and (d) BT2MGW.

Fig. 3a–d show the surface morphology of MGW, ATMGW, BT1MGW and BT2MGW, respectively. Generally, the SEM micrographs revealed that following the treatment processes, the cellulose fibres extracted from the MGW took on a fibrous structure. The surface morphology of ATMGW indicates the initial formation of fibres, resulting from the alkali treatment. The fibres were then observed to transform into thinner fibrils with a reticular configuration. This transformation, which is shown in Fig. 3c and d, suggests that the purification and bleaching procedures did not trigger the severing of cellulose fibres.

### TEM analysis

**Fig. 4:**
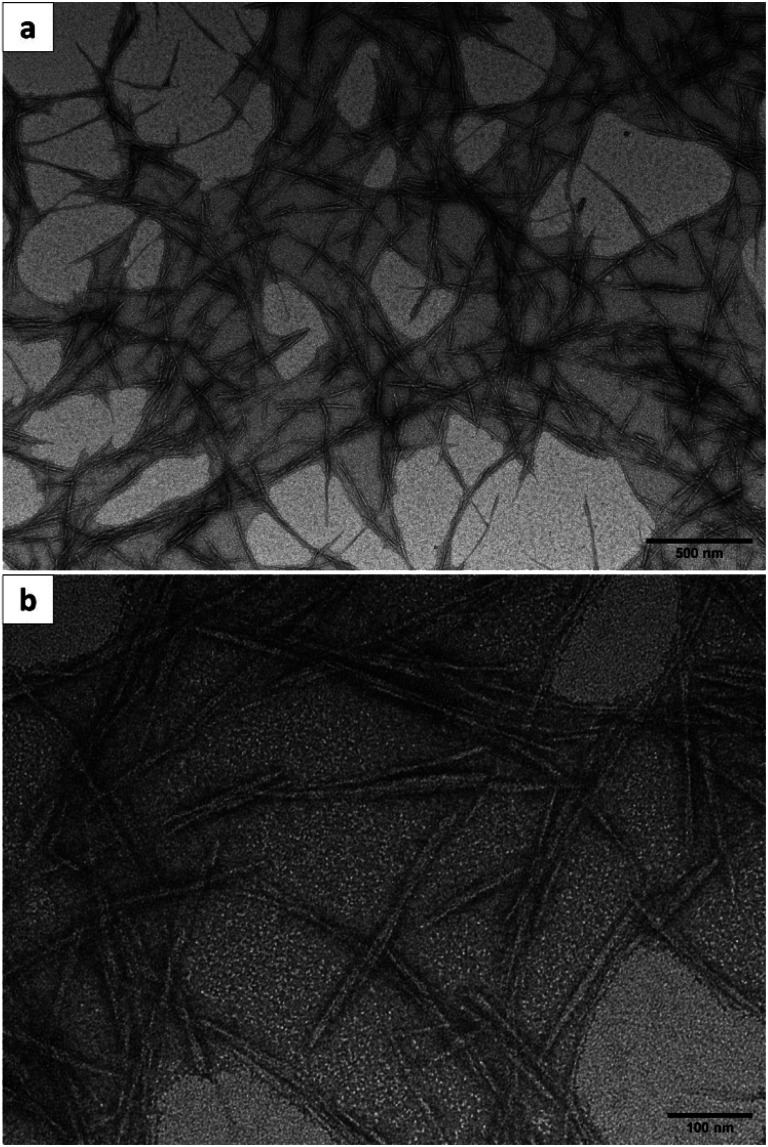
TEM images of CNCs produced from the municipal grass waste at (a) ×10k magnification and (b) ×40k magnification.

Fig. 4a and b show the TEM images of the CNCs produced from the MGW at different magnification. The morphology of the CNCs acquired through acid hydrolysis, led to their assuming a rod-like structure as depicted in Fig. 4a and b. The longitudinal arrangement of some agglomerated rods (Fig. 4a) may be attributed to the presence of hydrogen bonding. The independent, unconnected whiskers exhibited in Fig. 4b, are regular features of the CNCs. The development of these features stems from the high aspect ratio, the extensive surface area, and the presence of a profusion of hydroxyl groups on the surface. Such a situation facilitates the stacking or interacting of the nanoparticles, through van der Waals or hydrogen bonding interactions.^56^ Although this consequently culminates in agglomeration, dispersal is straightforward in an aqueous medium. Agglomeration can also result from water evaporation during the preparation of the samples.

**Fig. 5:**
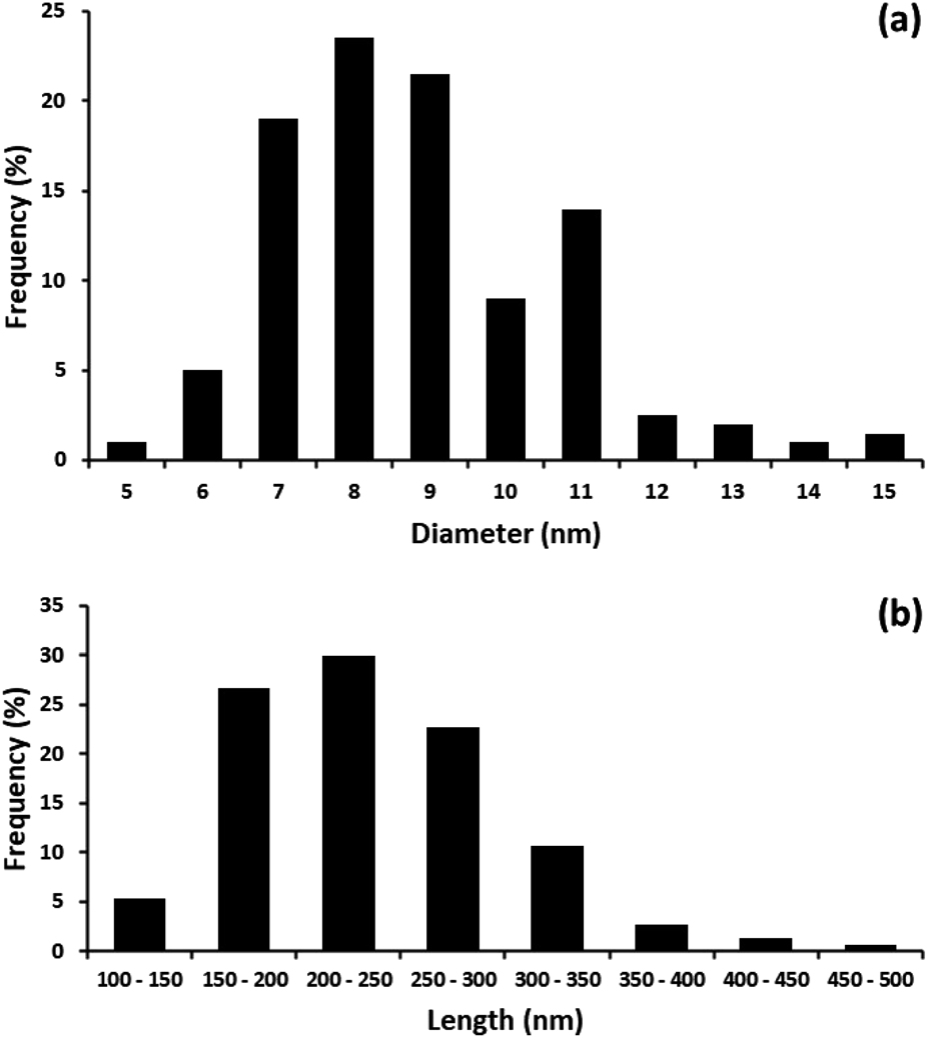
Distribution of (a) diameter and (b) length of CNCs.

The distribution of diameter and length of the CNCs produced from the MGW are shown in Fig. 5a and b, respectively. The diameter of the CNCs ranged from 5 to 15 nm while the length ranged from 100 to 500 nm. The average diameter and length of the CNCs were recorded as ∼8.4 nm and ∼236 nm respectively. These measurements are comparable to those documented in literature associated to CNCs. Briefly, the average diameter of the CNCs obtained is similar when compared to the value of CNCs produced from other agro-waste materials. For examples, the average diameter of the CNCs extracted from hemp is 15 nm,^32^ and 9.45 nm for CNCs obtained from sisal.^33^ The average diameter of CNCs extracted from rice straw^39^ and royal palm tree^45^ was found to be 5.95 nm and 8.2 nm, respectively. Table 1 provides a summary of the geometrical dimensions of CNCs, derived from a variety of source materials.

**Table 1:** Geometrical dimensions of CNCs prepared from various source material

Source material	Diameter (nm)	Length (nm)	CI (%)	Ref.
Municipal grass waste	5–15	100–500	58.2	This study
Wood pulps	17	190–660	n.a.	27
Cellulose powder	10–25	>1000	65.8	29
Microcrystalline cellulose	5	276	72.1	30
Cotton	10	133	82.0	31
Hemp	15	100–200	87	32
Sisal	9.45 ± 1.85	128.55 ± 20.51	68	33
Wastepaper	4.40 ± 3.91	356.27 ± 137.28	92.6	22
	3–10	100–300	75.9	23
Sugarcane bagasse	12 ± 1.6	222 ± 23	65	38
Rice straw	5.95	270	86	39
Apple pomace	7.9 ± 1.25	28 ± 2.03	78	41
Tomato peels	5–9	100–200	80.8	43
Royal palm tree	8.1 ± 2.5	112.9 ± 43.6	63.6	45

Irrespective of the sources of the precursors utilized, the production of similar geometrical CNCs, have also been achieved, by researchers utilizing unconventional approaches. For instance, an undertaking by Lee *et al.*^57^ involved the use of electron beam irradiation as the extraction method, to obtain CNCs with a consistent width of 23 to 31 nm, and a tuneable length of 128 to 747 nm. An acid-free preparation approach, involving TEMPO oxidation followed by cavitation, was employed by Zhou *et al.*^58^ to acquire CNCs with a consistent width of 3.5 to 3.6 nm, and an average length of 200 nm. Isolation of CNCs using high energy ball milling method showed rod-like morphology with length and diameter ranged between 200 and 400 nm and 7 to 18 nm, respectively.^59^

### XRD analysis

**Fig. 6:**
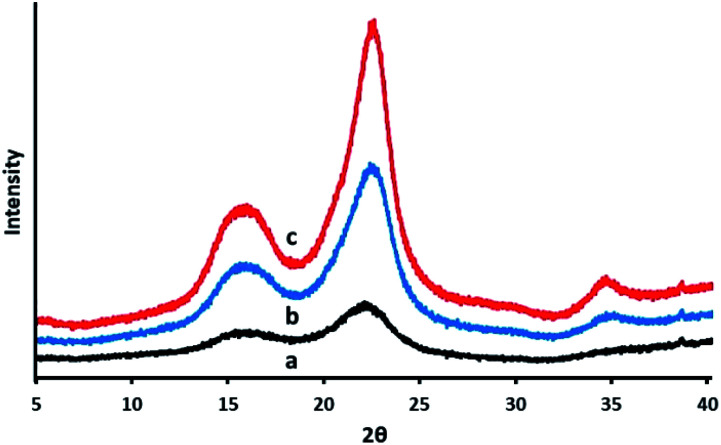
X-ray diffractogram of (a) PTMGW, (b) BT2MGW and (c) CNCs.

After the purification treatments, the CNCs were assessed for their crystallinity. The hydroxyl groups bring about the occurrence of intra, as well as intermolecular hydrogen bonding, in cellulose. This gives rise to a variety of ordered crystalline configurations. As displayed in Fig. 6, the XRD analysis exhibited two kinds of peaks. The peaks with the greatest intensity values of between 2*θ* = 22.3° corresponds to the crystalline structure of cellulose I, whilst the lower diffraction peak of 2*θ* in the range of 14° to 17°, are associated to amorphous background.^60,61^ The peak appearing at 34° is also associated to the presence of cellulose I.^62^ At 22.3°, the main peak associated to the crystalline structure of cellulose I became apparent in the CNCs, which suggest the presence of a crystalline material comprising nanoscale crystals, while the lower diffraction peak (2*θ* = 14–16°) represents the amorphous part.^61^ The presence of some form of amorphous material is also indicated by the background ‘hump’, appearing in the vicinity of 20° to 30°. The effectiveness of the acid hydrolysis treatment for the elimination of the amorphous region is made evident by the somewhat more distinct crystalline peak in the CNCs.

Eqn (1) was applied to approximate the average cross-sectional dimension of the elementary cellulose crystallites, through their X-ray diffractograms. This is explained in XRD Experimental section. Although the Scherrer equation used is undependable for dimensions less than 100 nm, it is nevertheless applicable for determining the approximate average crystallite size. As shown in Table 2, with the FWHM for the PTMGW, BT2MGW and CNCs revealed as 0.19, 0.38 and 0.22 respectively, the crystallite size was found to be 73.12, 29.52 and 42.79 nm. The crystallinity index (CI) listed in Table 2 indicates that the crystallinity of the CNCs was increased from 40.5% to 58.2%, through purification treatments and acid hydrolysis.

**Table 2:** Crystallinity of pretreated MGW, BT2MGW and CNCs

Sample	2*θ*_002_	*I*_002_	2*θ*_am_	*I*_am_	FWHM_002_	Average crystal size (nm)	CI (%)
PTMGW	22.2	1901.1	14.9	1130.3	0.19	73.12	40.5
BT2MGW	22.1	3447.8	15.2	1681.1	0.38	29.52	51.2
CNCs	22.3	3766.1	15.5	1573.8	0.22	42.79	58.2

The crystallinity index is lower when compared to other CNCs produced from agricultural waste, as shown in Table 1. Despite an optimum acid hydrolysis procedure might need to be further investigated, this can also be expected since the precursor used exhibited a lower crystallinity index (CI) of 40.5% as compared to other cellulose precursors. The low crystallinity of CNCs was also reported by others. For instance, Phanthong *et al.*^29^ reported the CI of 65.8% for CNCs produced from cellulose powder which having 73.7% crystallinity, whereby the crystallinity was reduced as compared to the precursor used. Besides, Li *et al.*^63^ and Han *et al.*^64^ reported the reduction of ∼30–34% crystallinity, from the cellulose precursor, which produce CNCs with 36% and 52.1% crystallinity, respectively.

However, it should be note that the increase of around 18% of the crystallinity was found in the present study. Besides, the increased percentage is similar to other reported literature such as 20% increased for CNCs produced from cotton,^31^ and 22% increased for CNCs prepared from sisal.^33^ Jiang & Hsieh^43^ and Danial *et al.*^23^ reported the increase of only 10–12% crystallinity for the CNCs produced from tomato peels cellulose and wastepaper, respectively. Lu & Hsieh^39^ reported that the crystallinity of the CNCs can be increased by increasing the hydrolysis time, which directly influence the crystallinity degree. The crystallinity is related to the strength and stiffness of the cellulose fibers.^65^ The importance of higher crystallinity index, however, will be dependent on CNCs application. Since the crystallinity affects the mechanical and physical properties,^66^ the high crystallinity CNCs might be useful as reinforcement in nanocomposites, while the low crystallinity CNCs can be indicated to be applied as a food thickener or pickering emulsion.^66^ Although the crystallinity might affect the strength and fiber stiffness, the presence of the cellulose surface functional groups also plays a significant role in determining the applicability and reinforcing behavior of the CNCs.

## Conclusions

This study reveals the viability of acquiring cellulose nanocrystals from municipal grass waste or grass clippings. The application of alkaline, bleaching, and acid hydrolysis treatments effectively isolated CNCs from the municipal grass waste. The efficiency of the treatment approach was verified through the FTIR analysis, while the nano-scale dimensions of the CNCs acquired were determined through the TEM analysis. The crystallinity value of the CNCs acquired was effectively raised from 40.5% to 58.2%, through purification treatments and acid hydrolysis. The CNCs obtained displayed the usual whisker- or rod-like feature, with an average dimension of 8.4 nm for diameter, and length ranging from 100 to 500 nm. Given the abundance and the need to reduce the municipal grass waste, this work demonstrated that the CNCs can be successfully obtained from which not only contributing as a new source of nanomaterials but also minimizing the environmental liability. Although the nanomaterial production are compensated with extensive use of chemicals from several treatment processes and further considerable investigation might be required for upscale production, this study might pave the way towards the valorization of alternative precursor for the production of CNCs without intermeddling with commonly used precursors such as cotton, wood pulps and wastepaper that already have their significant manufacturing cycle in production of cellulose or recycling industries.

## Conflicts of interest

There are no conflicts to declare.

## Supplementary Material

## References

[cit1] Habibi Y., Lucia L. A., Rojas O. J. (2010). Chem. Rev..

[cit2] Kontturi E., Laaksonen P., Linder M. B., Nonappa, Gröschel A. H., Rojas O. J., Ikkala O. (2018). Adv. Mater..

[cit3] Cao L., Liu C., Zou D., Zhang S., Chen Y. (2020). Carbohydr. Polym..

[cit4] Hynninen V., Mohammadi P., Wagermaier W., Hietala S., Linder M. B., Ikkala O., Nonappa (2019). Eur. Polym. J..

[cit5] Lavoine N., Bergström L. (2017). J. Mater. Chem. A.

[cit6] Menon M. P., Selvakumar R., Kumar P. S., Ramakrishna S. (2017). RSC Adv..

[cit7] Mariano M., El Kissi N., Dufresne A. (2014). J. Polym. Sci., Part B: Polym. Phys..

[cit8] Motaung T. E., Linganiso L. Z. (2018). Int. J. Plast. Technol..

[cit9] Kang X., Kuga S., Wang C., Zhao Y., Wu M., Huang Y. (2018). ACS Sustainable Chem. Eng..

[cit10] Kumar S., Falzon B. G., Kun J., Wilson E., Graninger G., Hawkins S. C. (2020). Composites, Part A.

[cit11] Liu X., Yang K., Chang M., Wang X., Ren J. (2020). Carbohydr. Polym..

[cit12] Shaghaleh H., Xu X., Wang S. (2018). RSC Adv..

[cit13] Du H., Liu W., Zhang M., Si C., Zhang X., Li B. (2019). Carbohydr. Polym..

[cit14] Yadav M., Chiu F.-C. (2019). Carbohydr. Polym..

[cit15] Mujica-Garcia A., Hooshmand S., Skrifvars M., Kenny J. M., Oksman K., Peponi L. (2016). RSC Adv..

[cit16] Karim Z., Mathew A. P., Kokol V., Wei J., Grahn M. (2016). RSC Adv..

[cit17] Bumbudsanpharoke N., Kwon S., Lee W., Ko S. (2019). Int. J. Biol. Macromol..

[cit18] Oyewo O. A., Mutesse B., Leswifi T. Y., Onyango M. S. (2019). J. Environ. Chem. Eng..

[cit19] Yu F., Zhou Y., Qiao H., Sun L., Li L., Feng C., Li Y. (2016). RSC Adv..

[cit20] Gong J., Li J., Xu J., Xiang Z., Mo L. (2017). RSC Adv..

[cit21] Trache D., Hussin M. H., Haafiz M. K. M., Thakur V. K. (2017). Nanoscale.

[cit22] Campano C., Miranda R., Merayo N., Negro C., Blanco A. (2017). Carbohydr. Polym..

[cit23] Danial W. H., Abdul Majid Z., Mohd Muhid M. N., Triwahyono S., Bakar M. B., Ramli Z. (2015). Carbohydr. Polym..

[cit24] Hanafiah S. F. M., Danial W. H., Samah M. A. A., Samad W. Z., Susanti D., Salim R. M., Majid Z. A. (2019). Malaysian J. Anal. Sci..

[cit25] Orue A., Santamaria-Echart A., Eceiza A., Peña-Rodriguez C., Arbelaiz A. (2017). J. Appl. Polym. Sci..

[cit26] Peretz R., Sterenzon E., Gerchman Y., Kumar Vadivel V., Luxbacher T., Mamane H. (2019). Carbohydr. Polym..

[cit27] Dong H., Strawhecker K. E., Snyder J. F., Orlicki J. A., Reiner R. S., Rudie A. W. (2012). Carbohydr. Polym..

[cit28] Dong S., Bortner M. J., Roman M. (2016). Ind. Crops Prod..

[cit29] Phanthong P., Karnjanakom S., Reubroycharoen P., Hao X., Abudula A., Guan G. (2017). Cellulose.

[cit30] Di Giorgio L., Martín L., Salgado P. R., Mauri A. N. (2020). Carbohydr. Polym..

[cit31] Hemmati F., Jafari S. M., Taheri R. A. (2019). Int. J. Biol. Macromol..

[cit32] Luzi F., Fortunati E., Puglia D., Lavorgna M., Santulli C., Kenny J. M., Torre L. (2014). Ind. Crops Prod..

[cit33] Mariano M., Cercená R., Soldi V. (2016). Ind. Crops Prod..

[cit34] Bhange V. P., William S. P. M. P., Vaidya A. N., Chokhandre A. R. (2012). Int. J. Recent Trends Sci. Technol..

[cit35] Bustamante M. A., Ceglie F. G., Aly A., Mihreteab H. T., Ciaccia C., Tittarelli F. (2016). J. Environ. Manage..

[cit36] López M., Soliva M., Martínez-Farré F. X., Bonmatí A., Huerta-Pujol O. (2010). Bioresour. Technol..

[cit37] Hernández-Gómez A., Calderón A., Medina C., Sanchez-Torres V., Oviedo-Ocaña E. R. (2020). Environ. Sci. Pollut. Res..

[cit38] Leão R. M., Miléo P. C., Maia J. M. L. L., Luz S. M. (2017). Carbohydr. Polym..

[cit39] Lu P., Hsieh Y.-L. (2012). Carbohydr. Polym..

[cit40] Wibowo A., Madani H., Judawisastra H., Restiawaty E., Lazarus C., Budhi Y. W. (2018). IOP Conf. Ser. Earth Environ. Sci..

[cit41] Melikoğlu A. Y., Bilek S. E., Cesur S. (2019). Carbohydr. Polym..

[cit42] Sai Prasanna N., Mitra J. (2020). Carbohydr. Polym..

[cit43] Jiang F., Hsieh Y.-L. (2015). Carbohydr. Polym..

[cit44] Madureira A. R., Atatoprak T., Çabuk D., Sousa F., Pullar R. C., Pintado M. (2018). Int. J. Food Stud..

[cit45] Hafemann E., Battisti R., Marangoni C., Machado R. A. F. (2019). Carbohydr. Polym..

[cit46] de Assis C. A., Houtman C., Phillips R., Bilek E. M. T., Rojas O. J., Pal L., Peresin M. S., Jameel H., Gonzalez R. (2017). Biofuels, Bioprod. Biorefin..

[cit47] Candido R. G., Gonçalves A. R. (2016). Carbohydr. Polym..

[cit48] Segal L., Creely J. J., Martin A. E., Conrad C. M. (1959). Text. Res. J..

[cit49] Tserki V., Zafeiropoulos N. E., Simon F., Panayiotou C. (2005). Composites, Part A.

[cit50] Roncero M. B., Torres A. L., Colom J. F., Vidal T. (2005). Bioresour. Technol..

[cit51] Johar N., Ahmad I., Dufresne A. (2012). Ind. Crops Prod..

[cit52] Yan X., Cheng J.-R., Wang Y.-T., Zhu M.-J. (2020). Bioresour. Technol..

[cit53] Garside P., Wyeth P. (2003). Stud. Conserv..

[cit54] Li Y., Liu Y., Chen W., Wang Q., Liu Y., Li J., Yu H. (2016). Green Chem..

[cit55] Cherian B. M., Leão A. L., de Souza S. F., Thomas S., Pothan L. A., Kottaisamy M. (2010). Carbohydr. Polym..

[cit56] Lu H., Gui Y., Zheng L., Liu X. (2013). Food Res. Int..

[cit57] Lee M., Heo M. H., Lee H., Lee H.-H., Jeong H., Kim Y.-W., Shin J. (2018). Green Chem..

[cit58] Zhou Y., Saito T., Bergström L., Isogai A. (2018). Biomacromolecules.

[cit59] Mohd Amin K. N., Annamalai P. K., Morrow I. C., Martin D. (2015). RSC Adv..

[cit60] Cao X., Ding B., Yu J., Al-Deyab S. S. (2012). Carbohydr. Polym..

[cit61] French A. D., Santiago Cintrón M. (2013). Cellulose.

[cit62] Asempour F., Emadzadeh D., Matsuura T., Kruczek B. (2018). Desalination.

[cit63] Li J., Wei X., Wang Q., Chen J., Chang G., Kong L., Su J., Liu Y. (2012). Carbohydr. Polym..

[cit64] Han J., Zhou C., French A. D., Han G., Wu Q. (2013). Carbohydr. Polym..

[cit65] Yao W., Weng Y., Catchmark J. M. (2020). Cellulose.

[cit66] de Souza A. G., Junqueira M. T., de Lima G. F., Rangari V. K., Rosa D. S. (2020). J. Polym. Environ..

